# Different reliability of instrumented gait analysis between patients with unilateral hip osteoarthritis, unilateral hip prosthesis and healthy controls

**DOI:** 10.1186/s12891-018-2145-0

**Published:** 2018-07-18

**Authors:** Roland Zügner, Roy Tranberg, Vera Lisovskaja, Johan Kärrholm

**Affiliations:** 1 Department of Orthopaedics, Institute of Clinical Sciences, Sahlgrenska Academy, University of Gothenburg, Sahlgrenska University, 413 45 Göteborg, SE Sweden; 2 Department of Economics, Institute of Communication in Statistics, University of Gothenburg, Gothenburg, Sweden; 3 Lundberg Laboratory for Orthopaedic Research, Sahlgrenska University Hospital/Mölndal, Göteborgsvägen 31, SE-431 80 Mölndal, Sweden

**Keywords:** Gait analysis, Hip osteoarthritis, Hip prosthesis, Healthy controls, Inter-observer reliability, Rehabilitation

## Abstract

**Background:**

The gait pattern varies within the population and between patient groups with different musculoskeletal diseases. It also varies over time due to various reasons. Three-dimensional gait analysis (3DGA) is frequently used to measure these changes, but the precision of this methodology may vary.

**Methods:**

We primarily aimed to study the repeatability of hip motion measurements in patients with unilateral osteoarthritis (OA), patients with unilateral total hip arthroplasty (THA) and healthy controls. A secondary aim was to delineate any differences in hip motion during walking between these groups. Ten males and 10 females in each group were recruited. All patients underwent gait assessments using 3DGA recorded by 2 examiners. Data was analysed with comparison of variance and linear regression.

**Results:**

The variability of the extension-flexion recordings was smallest in healthy controls (SD < 7.7°), increased in patients with THA (SD < 11.1°) and was most pronounced in the OA patients (SD < 12.2°). The degree of hip extension-flexion turned out to be the variable that most effectively could separate the controls from the 2 patient groups and the patient groups from each other. One to 2 years after THA the gait pattern was improved but still differed comparing a group of THA from a group of healthy controls.

**Conclusions:**

Patients with hip osteoarthritis showed the poorest repeatability between gait recordings collected by different examiners, as compared to patients operated with a THA and healthy controls. The walking pattern after THA still differed from healthy controls 1–2 years after the operation.

## Background

Objective recordings of gait pattern are frequently used to document the influence of hip disease on the walking pattern both before and after treatment with total hip arthroplasty (THA). Evaluation of these recordings requires knowledge about measurement errors and individual variability, which could vary between different diagnoses of musculoskeletal diseases [1].

Several studies used three-dimensional gait analysis (3DGA) to differentiate motion patterns between groups of subjects or to detect any correlation between gait patterns and self-reported health parameters [2–5]. Ewen et al. reviewed seven articles studying gait after total hip arthroplasty (THA). Three of them reported significantly decreased walking speed and decreased peak hip abduction moment as compared to controls. Furthermore, four studies found that the stride length as well as the range of hip extension-flexion was significantly lower. The authors pointed out that velocity, stride length, range of hip extension-flexion and peak abduction moment might differentiate a population of THA from controls [6–9]. Ornetti et al. (2010) made a systematic review of 3DGA studies on subjects with hip and knee osteoarthritis (OA). They concluded that there still is lack of validated and reliable kinematic data that can be used to distinguish between normal subjects and subjects with OA [10]. Recently, Laroche et al. carried out a test-retest study of 23 subjects with hip OA. They concluded that 2 to 10 gait trials are necessary to level out intrinsic variability, mainly depending on the parameters studied [11, 12].

Several authors have studied the reliability of 3DGA and a number of test-retest studies have reported methodological errors, based on repeated studies of individuals by different examiners. Most of these studies found that factors such as number of examiners, type of testing protocols and biomechanical models used could influence the results. It has also been reported that there is a greater variability among subjects with specific diagnoses. This indicates that it is essential to understand and quantify the sources of error in the 3DGA analyses within and between sessions, as well as between assessors, in order to obtain a correct interpretation of results.

Gait analysis performed on patient groups with a specific disease is commonly compared with healthy persons that serve as controls. Healthy controls are also frequently used to evaluate the reproducibility of the recordings. Patients who might suffer from diseases with a more severe influence on mobility might display a less consistent gait pattern due to various reasons such as joint deformity, limping that may vary over time, pain or neuromuscular disease. For such patient groups a higher variability of the recorded kinematics and kinetics might be present between sessions, which will have implications on the resolution of the recordings when patient groups with different types of diseases are compared [10, 11, 13–16].

## Methods

### Aim

We studied the gait pattern using 3DGA in 3 groups; healthy controls, subjects with unilateral hip OA and subjects operated with unilateral THA. Each of the subjects was examined by 2 observers. The primary aim of the study was to determine whether there is a systematic difference concerning repeatability of measurements within subjects with- or without hip disease, or with a replaced hip joint in terms of hip kinematic and kinetic data obtained from the 3DGA measurements. The secondary aim was to delineate differences in hip motion during walking between these groups.

For data acquisition, a 12-camera motion capture system with a sampling rate of 240 Hz (Oqus 4, Qualisys AB, Göteborg, Sweden) together with 2 force-plates (Kistler 9182C, Kistler Group, Winterthur, Switzerland) were used.

### Participants

This cross-sectional test-retest study included 3 groups with 20 subjects in each group (Table 1). The first group constituted healthy controls, the second group subjects with unilateral hip OA and the third group subjects operated with unilateral THA. Gender was equally distributed throughout the groups (Table 1). The control group was recruited locally from laboratory staff and their relatives and friends. None of the healthy subjects had any problems related to the musculoskeletal system.

**Table 1:** Distribution of age and BMI for the 10 males and 10 females in each group (Healthy, Hip osteoarthritis-OA, Total Hip arthroplasty-THA)

	Healthy						OA						THA					
	Male			Female			Male			Female			Male			Female		
	Mean	SD	95% CI	Mean	SD	95% CI	Mean	SD	95% CI	Mean	SD	95% CI	Mean	SD	95% CI	Mean	SD	95% CI
Age	45	23	37.5–52.6	46	17	40.7–51.9	55	21	45.2–64.8	62	7.8	58.7–66.1	61	15	54.1–68.3	65	12.3	59.1–70.7
BMI	24.4	1.5	23.7–25.1	24.3	2.9	23–25.7	29.4	2.7	28.2–30.7	26.6	5.9	23.8–29.4	31.4	6.0	28.5–34.2	27.3	5.4	24.8–29.8

Mean, standard deviation (*SD*) and 95% confidence interval of the mean (*95% CI*) are presented

Subjects with hip OA were recruited from the waiting list for hip surgery at the Department of Orthopaedics at our hospital. Presence of hip OA was verified on radiographs. Six hips were classified as Stage 2 according to Ahlbäck, 10 hips as Stage 3 and 4 hips as Stage 4 [17]. On the contralateral side, all subjects were without symptoms. Twelve had no signs of OA and 8 had a minor reduction of the joint space (Stage 1). All radiographs were re-evaluated by one of the senior authors with about 40 years’ experience in the field of orthopedics and with special interest in total hip and knee replacement*.*

All 20 subjects with unilateral THA had undergone surgery 1–2 years prior to the study. Thirteen of these subjects had their surgery on their right side. Femoral head sizes of 32 mm (18 hips), 36 mm (1 hip) and 28 mm (1 hip) had been used. A lateral incision was used in 13 hips, and an anterior incision in 3 hips. For the remaining 4 hips, a posterior incision was used. All subjects were without symptoms on the contra lateral side, even though radiographs revealed that 7 subjects had minor reduction of the joint space (Stage 1) [17].

Two examiners with more than 15 years of experience in 3DGA examined all subjects. In order to record the hip kinematic with the 3DGA a number of 15 spherical markers (∅ 11 mm) were attached to the skin of the lower extremities and the pelvis according to the modified Helen Hays model. This was made with double-adhesive tape according to a skin marker model presented in detail by Weidow et al. [18–20]. For the skin marker model, markers were attached to the proximal boarder of sacrum, anterior/superior of iliac spine, lateral knee joint line, proximal boarder of patella, tibial tubercle, tuber calcanei at the heel, lateral malleolus, and finally between the second and third metatarsals. A modified Coda pelvis was used in the marker model. This segment was based on the bilateral markers on anterior superior iliac spine together with one marker on the mid-point on the proximal border of sacrum. Hip-joint centres were defined in relation to the pelvis segment, according to recommendations of Bell et al. for right and left hip-joint centres [19, 20].

Each examiner applied all markers before each of the examinations and recorded the data. The order in which the 2 examiners studied the subjects was randomized. Both examinations were performed during the same session within a 2-h period. In total 120 examinations were performed. During the examination and recording of data the subjects were wearing underwear and subjects were first asked to walk 5–10 times in a self-selected speed through the calibrated volume to familiarize with the situation. Then 6 approved gait-trials were recorded and 1 trial was randomly selected for further examination.

For calculations of kinematic and kinetic peak variables together with spatiotemporal gait parameters, the Visual 3D™ software (C-Motion, Inc., Germatown, USA) was used.

### Statistics

Two analyses were performed. In the first analysis, we determined the magnitude of differences of data recorded between the two 2 observers for each parameter of interest in the three patient groups. The variances for these differences were calculated for each group and their equality was assessed by means of Bartlett’s test (Table 3).

In the second analysis we evaluated the systematic differences in group joint kinematics and kinetics using ANOVA including data from all 3 groups and linear regression for pairwise comparison between groups. In the first linear regression model only the membership was included as dependent variable (Table 4), while in the second one speed, BMI and age were added to the covariates to compensate for any differences in BMI and age between the 3 groups (Table 5). Such a model can also be seen as an ANCOVA. In these analyses the average values between the 2 examiners were used. The confidence intervals were calculated using normal approximations.

**Fig. 1:**
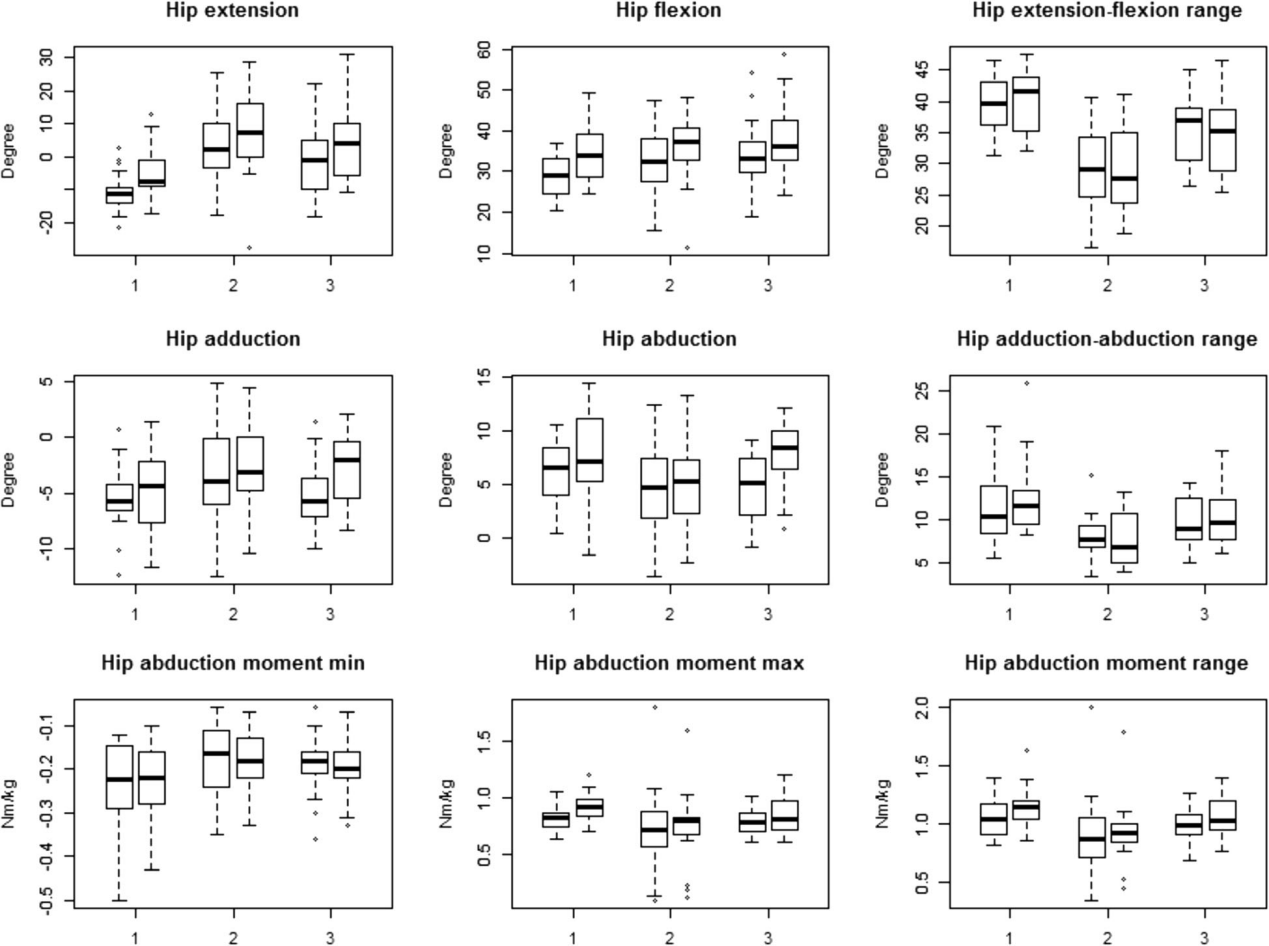
Box-plots of hip motions. Box-plots of hip motions (line 1–2) and moments (line 3) in healthy controls (labelled 1), patients with hip osteoarthritis (2) and with unilateral THR (3), male vs. female. Top: extension, flexion and range of extension-flexion in degrees. Middle: adduction, abduction and range of adduction-abduction in degrees. Bottom: abduction, adduction and range of abduction-adduction moments in Nm/kg

Bland-Altman plots for 3DGA joints kinematics of hip extension-flexion, adduction-abduction and joint moments of adduction-abduction were constructed after averaging between the 2 examiners. Data are also illustrated with box plots (Fig. 1).

The affected side was investigated in the OA and THA subjects and only the right side in the healthy subjects.

## Results

### Analysis of variance

**Fig. 2:**
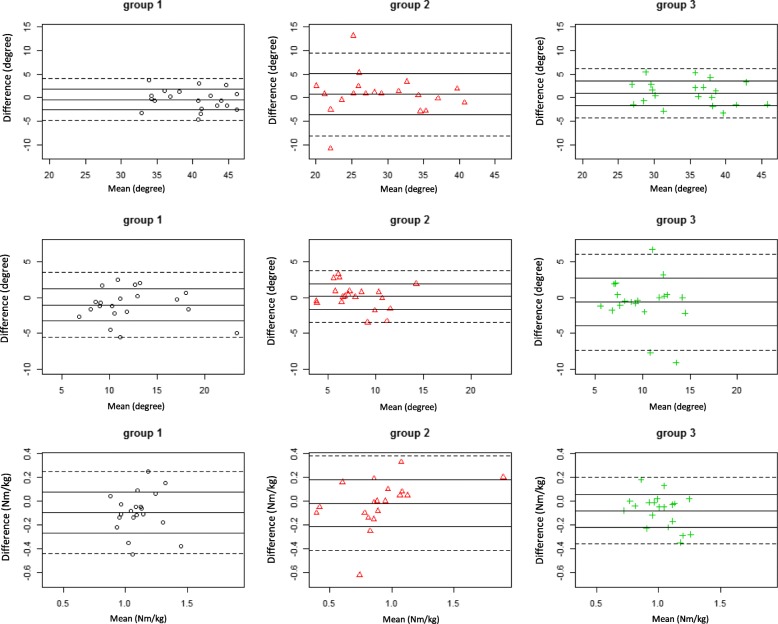
Bland-Altman plots of mean values. Bland-Altman plots of mean values versus difference between examiner 1 and 2 are shown for selected parameters in healthy controls (group 1), patients with hip osteoarthritis (group 2) and with unilateral THR (group 3). Top: range of hip extension-flexion in degrees. Middle: range of adduction-abduction in degrees. Bottom: range of abduction-adduction moment in Nm/kg. The five lines in each plot, indicate 1) the mean of the between-observer difference 2) mean plus/minus one SD 3) mean plus/minus two SD

For most of the variables studied, the data scatter between examiner 1 and 2 was of similar magnitude in the 3 groups (Fig. 1, Table 2). The differences between the groups with regards to measurement precision reached significance in the comparisons between groups for range of hip extension-flexion (Healthy vs. OA, *p* ≤ 0.005, 2.2°; OA vs THA, *p* ≤ 0.027, 2.6°), range of hip abduction-adduction (OA vs THA, *p* ≤ 0.01, 3.4°) and hip abduction moment (OA vs THA, *p* ≤ 0.02, Table 3). Plots of the range in kinematic and kinetics mean values versus difference between examiner 1 and 2 (Bland-Altman plots) are shown for the hip joint extension-flexion, adduction-abduction and kinetics for adduction-abduction moment (Fig. 2).

**Table 2:** Speed, hip kinematics in the sagittal and frontal planes and hip kinetics in the frontal plane, for the 10 males and 10 females in each group, as recorded by the 2 examiners

	Healthy, examiner 1		Healthy, examiner 2		OA, examiner 1		OA, examiner 2		THA, examiner 1		THA, examiner 2	
	Mean	95% CI	Mean	95% CI	Mean	95% CI	Mean	95% CI	Mean	95% CI	Mean	95% CI
Speed *m/s*	1.18	1.1 to 1.2	1.19	1.1 to 1.3	0.97	0.88 to 1.1	0.95	0.84 to 1.1	1.1	1 to 1.2	1.1	0.99 to 1.2
Hip extension *degrees*	−10.8	−13.6 to −8.0	−5.2	−8.8 to −1.6	3.4	−1.9 to 8.6	7.0	1.2 to 12.7	−1.6	−6.5 to 3.3	3.3	−1.9 to 8.5
Hip flexion *degrees*	28.8	26.5 to 31.1	34.9	31.3 to 38.5	32.7	29 to 36.4	35.7	31.7 to 39.6	33.8	30 to 37.7	37.8	33.6 to 41.9
Hip ext-flex range *degrees*	39.7	37.6 to 41.7	40.1	37.9 to 42.3	29.3	26.2 to 32.5	28.7	25.6 to 31.9	35.4	32.9 to 38	34.5	31.7 to 37.3
Hip adduction *degrees*	−5.5	−7.0 to −4.1	−5.0	−6.8 to −3.2	−3.4	− 5.5 to −1.3)	−2.5	−4.3 to −0.6)	−5.0	−6.5 to −3.5)	−2.6	− 4.1 to − 1.2)
Hip abduction *degrees*	6.1	4.8 to 7.4	7.6	5.7 to 9.4	4.5	2.7 to 6.3	5.5	3.6 to 7.3	4.7	3.2 to 6.2	7.7	6.3 to 9.1
Hip add-abd range *degrees*	11.6	9.6 to 13.5	12.5	10.6 to 14.5	7.9	6.6 to 9.2	7.9	6.5 to 9.4	9.7	8.4 to 11.1	10.4	8.8 to 11.9
Hip add moment *Nm/kg*	0.82	0.77 to 0.86	0.9	0.85 to 0.97	0.7	0.54 to 0.87	0.74	0.58 to 0.90	0.79	0.73 to 0.85	0.86	0.78 to 0.94
Hip abd moment *Nm/kg*	−0.24	−0.28 to −0.19	−0.23	−0.27 to − 0.19	−0.2	− 0.22 to − 0.14)	−0.18	− 0.21 to − 0.14)	−0.19	− 0.22 to − 0.16)	−0.19	− 0.20 to − 0.16)
Hip add-abd moment range *Nm/kg*	1.05	0.97 to 1.1	1.1	1.06 to 1.2	0.9	0.73 to 1.05	0.9	0.77 to 1.1	0.98	0.91 to 1.04	1.05	0.97 to 1.15

Mean and 95% confidence interval of mean (*CI*) in Healthy (*H*), Hip osteoarthritis (*OA*) and Total hip arthroplasty (*THA*) subjects

**Table 3:** Standard deviations differences for the two examiners for each of the parameters presented in Table 2

	Standard deviations differences			H vs. OA	H vs. THA	OA vs. THA
	Healthy	OA	THA	*p*-value	p-value	*p*-value
Speed m/s	0.08	0.12	0.07	0.142	0.660	0.058
Hip extension degrees	8.63	9.55	8.67	0.664	0.984	0.678
Hip flexion degrees	8.51	9.77	9.50	0.557	0.638	0.907
Hip ext-flex range degrees	2.22	4.37	2.58	**0.005**	0.521	**0.027**
Hip adduction degrees	3.09	2.92	3.94	0.807	0.298	0.200
Hip abduction degrees	3.46	3.31	3.60	0,844	0.872	0.720
Hip add-abd range degrees	2.27	1.81	3.35	0.339	0.097	**0.010**
Hip add moment Nm/kg	0.14	0.19	0.11	0.152	0.143	0.974
Hip abd moment Nm/kg	0.08	0.05	0.05	0.138	0.391	**0.021**
Hip add-abd moment range Nm/kg	0.17	0.20	0.14	0.555	0.352	0.131

P-values refers to comparison of variances (Bartlett’s test) between Healthy (*H*), Hip osteoarthritis (*OA*) and Total hip arthroplasty (*THA*) subjects

### Analysis of association

The linear regression modelling using only group as covariate showed that patients with hip OA compared to healthy controls had slower walking speed, reduced hip extension and extension-flexion range, reduced range of adduction-abduction, reduced adduction moment and range of adduction-abduction moment (*p* ≤ 0.04, Table 4). After inclusion of the covariates speed, age and BMI only reduced hip extension and range of hip extension-flexion were still remaining (*p* ≤ 0.03, Table 5).

**Table 4:** Linear models using only group as covariate comparing healthy controls (H), patients with hip osteoarthritis (OA) and with total hip arthroplasty (THA)

	ANOVA linear model	Linear regression					
	All 3 groups	H vs OA		H vs THA		OA vs THA	
	p-value	Estimate* (95% C.I.)	p-value	Estimate* (95% C.I.)	p-value	Estimate* (95% C.I.)	p-value
Speed m/s	**0.001**	−0.23 (− 0.34 to − 0.12)	**< 0.0005**	− 0.09 (− 0.20 to 0.03)	0.15	0.14 (0.01 to 0.27)	**0.035**
Hip extension degree	**< 0.0005**	13.33 (7.83 to 18.82)	**< 0.0005**	8.86 (3.75 to 13.97)	**0.001**	−4.47 (−11.15 to 2.22)	0.18
Hip flexion degree	0.14	2.54 (−1.20 to 6.27)	0.18	3.98 (0.08 to 7.87)	**0.046**	1.44 (− 2.97 to 5.85)	0.51
Hip ext./flex range degree	**< 0.0005**	−10.8 (− 14.28 to − 7.32)	**< 0.0005**	−4.91 (−8.12 to − 1.69)	**0.004**	5.90 (2.09 to 9.70)	**0.003**
Hip adduction degree	0.10	2.18 (0.06 to 4.42)	0.056	1.40 (−0.36 to 3.16)	0.12	− 0.78 (− 2.85 to 1.29)	0.45
Hip abduction degree	0.12	−2.0 (−4.09 to 0.10)	0.06	−0,68 (− 2.46 to 1.11)	0.45	1.32 (− 0.65 to 3.29)	0.18
Hip add/abd range degree	**0.001**	−4.18 (−6.42 to − 1.94)	**0.001**	− 2.07 (− 4.30 to 0.17)	0.07	2.11 (0.39 to 3.84)	**0.02**
Hip adduction moment Nm/kg	0.07	0.05 (0.002 to 0.10)	**0.04**	0.04 (−0.007 to 0.09)	0.09	−0.01 (− 0.05 to 0.03)	0.59
Hip abduction moment Nm/kg	0.09	−0.15 (− 0.31 to 0.008)	0.06	− 0.05 (− 0.12 to 0.03)	0.23	0.10 (− 0.06 to 0.27)	0.21
Hip add/abd moment range Nm/kg	**0.02**	−0.20 (− 0.36 to − 0.05)	**0.01**	− 0.09 (− 0.18 to 0.009)	0.08	0.12 (− 0.04) to 0.27)	0.14

*P*-values for the overall comparison (ANOVA), p-values and estimates for the comparison between groups are presented (Linear regression)

*Beta-value

**Table 5:** Linear models including group, speed, BMI and age as covariate comparing healthy controls (H), patients with hip osteoarthritis (OA) and with unilateral total hip arthroplasty (THA)

	ANOVA linear model	Linear regression					
	All 3 groups	H vs OA		H vs THA		OA vs THA	
	*p*-value	Estimate* (95% C.I.)	*p*-value	Estimate* (95% C.I.)	*p*-value	Estimate* (95% C.I.)	*p*-value
Hip extension degree	**< 0.001**	6.18 (0.68 to 11.68)	**0.029**	6.47 (0.49 to 12.45)	**0.035**	0.65 (−5.76 to 7.07)	0.84
Hip flexion degree	0.706	−0.95 (− 5.40 to3.50)	0.67	1.98 (− 2.91 to 6.85)	0.42	3.27 (−1.32 to 7.85)	0.16
Hip ext./flex range degree	**< 0.001**	−7.15 (−10.48 to − 3.81)	**< 0.001**	− 4.53 (− 7.47 to − 1.60)	**0.003**	2.60 (− 0.81 to 6.00)	0.13
Hip adduction degree	0.356	2.11 (− 0.83 to 5.04)	0.15	1.44 (− 0.68 to 3.55)	0.17	− 1.11 (− 3.54 to 1.32)	0.36
Hip abduction degree	0.065	− 0.38 (−2.92 to 2.17)	0.78	− 0.21 (− 2.46 to 2.03)	0.85	0.29 (− 1.76 to 2.34)	0.77
Hip add/abd range degree	**0.001**	−2.47 (−5.21 to 0.28)	0.076	−1.62 (− 4.16 to 0.90)	0.20	1.40 (− 0.37 to 3.18)	0.12
Hip adduction moment Nm/kg	0.170	−0.16 (− 0.03 to 0.04)	0.11	− 0.08 (− 0.009 to 0.10)	0.10	0.11 (− 0.009 to 0.07)	0.25
Hip abduction moment Nm/kg	**0.003**	0.03 (−0.37 to 0.04)	0.34	0.05 (−0.18 to 0.02)	0.10	0.03 (−0.08 to 0.30)	0.13
Hip add/abd moment range Nm/kg	**0.041**	−0.19 (− 0.40 to − 0.007)	0.058	−0.12 (− 0.24 to − 0.007)	**0.039**	0.08 (− 0.10 to 0.26)	0.37

*P*-values for the overall comparison (ANOVA), *p*-values and estimates for the comparison between groups are presented (Linear regression)

*Beta-value

A corresponding comparison between patients with THA and controls revealed reduced hip extension, hip flexion and range of flexion-extension in the former group (*p* ≤ 0.046). After inclusion of the 3 covariates reduced hip extension, range of flexion-extension and range of hip adduction-abduction moment turned out to have significant influence (*p* < 0.04, Table 5).

Patients with OA walked more slowly than those who had been operated with a THA (*p* = 0.04). They had also reduced range of hip flexion-extension (*p* = 0.003) and range of adduction-abduction (*p* = 0.02). After inclusion of speed, age and BMI in the statistical model no differences remained.

## Discussion

Our primary purpose was to investigate if the variances of the 3DGA data differed depending on the status of the hip joint (presence of OA, operation with THA, normal hip joint). For most of the parameters studied, the data scatter turned out to be rather similar. In 4 of the comparisons, however, a significant difference could be detected (range of hip extension-flexion: OA vs. Healthy, OA vs. THA; range of adduction-abduction: OA vs. THA; abduction moment: OA vs. THA). The higher variances observed in the OA group could be caused by presence of pain in these patients which also has been noticed in knee osteoarthritis patients [21, 22]. The degree of pain might have varied in intensity between examinations. This reason and also muscular weakness or fatigue might have caused temporal changes of the walking pattern.

Another factor to consider is the soft tissue artefacts (STA) which have been studied earlier [23]. Peters et al. (2010) performed a systematic review including 20 articles with the intention to quantify soft tissue artefacts during 3DGA. In 11 of these studies invasive methods were used. The authors concluded that there are several important factors such as location of markers, activity performed, segment used and individual factors that influence the results. Soft tissue artefacts at the thigh up to 40 mm could occur and these authors called for improved methods to increase the resolution [24–26]. If such artefacts vary depending on the condition of the hip is not known, but the conditions of the soft tissues before and about 2 years after THA may change due to increasing physical activity after the operation.

Thus, the difference between examiners could be an effect of gait variations caused by intermittent pain and/or muscular fatigue in the OA group, but could also indicate difficulties to obtain reproducible marker placements when several examiners are involved [11, 13, 16, 23, 24, 27–29]. If so, a limited number of examiners, and preferably only the same ones should if possible, study a group of subjects scheduled for multiple follow-up occasions.

Regarding systematic differences between the 3 groups, it is well known from several studies [6, 7, 14, 30] that OA patients not only lose their ability to extend the hip during gait, but also their ability to produce a reasonable high abduction moment, resulting in a limping gait. The reduction in range of motion in the THA patients, particularly the hip extension, could be caused by persistent effects of the osteoarthritis such as soft-tissue contracture and muscle weakness, resulting in a remaining change of postural stability as compared to the uninvolved hip [31, 32]. Other reasons may include feelings of joint instability and the quality of the interfaces around the implant components, all potential factors that might influence the gait [33]. To what extent the hip implant conforms to the original anatomy of the hip is also of importance. Finally, it might be that the gait pattern developed during the disease period restitutes postoperatively only to a comfort level, corresponding to the requirements of the individual activities of daily living. There is no consensus concerning when the rehabilitation after a THA operation has reached a steady state. It can however, be theorised that the studied subjects in this study had reached this level when they were investigated, approximately 2 years after the operation. To confirm this theory a long time follow-up is needed.

One of the major limitations of this study is that the sample size is quite small, partly because of difficulties to recruit subjects with unilateral OA. Even though all subjects in our study had claimed that they had no symptoms on the contralateral side, a few of them had minor radiographic changes, implicating a presence of a mild osteoarthritis [17]. Furthermore, the small but significant differences regarding age, height, weight and BMI could have skewed the results.

## Conclusion

We found that the repeatability of hip extension-flexion varied between patient groups and was poorest in subjects with hip osteoarthritis. As noted previously, we also found that patients operated with a total hip arthroplasty tended to normalize their walking pattern, but still differed from healthy controls 2 years after the operation. Further studies including long-term follow up are desirable to evaluate if this walking pattern has become permanent or not.

## Abbreviations

3DGA: Three-dimensional gait analysis
BMI: Body mass index
OA: Osteoarthritis
STA: Soft tissue artefacts
THA: Total hip arthroplasty
